# An Integrated Analysis of the Response of Colorectal Adenocarcinoma Caco-2 Cells to X-Ray Exposure

**DOI:** 10.3389/fonc.2021.688919

**Published:** 2021-06-03

**Authors:** Isabella Guardamagna, Leonardo Lonati, Monica Savio, Lucia A. Stivala, Andrea Ottolenghi, Giorgio Baiocco

**Affiliations:** ^1^ Laboratory of Radiation Biophysics and Radiobiology, Department of Physics, University of Pavia, Pavia, Italy; ^2^ Immunology and General Pathology Unit, Department of Molecular Medicine, University of Pavia, Pavia, Italy

**Keywords:** Caco-2, ionizing radiation, cell survival and death, cell cycle, metalloproteases, G_2_/M arrest, genomic aberrations

## Abstract

Colorectal cancer is among the three top cancer types for incidence and the second in terms of mortality, usually managed with surgery, chemotherapy and radiotherapy. In particular, radiotherapeutic concepts are crucial for the management of advanced rectal cancer, but patients’ survival remains poor, despite advances in treatment modalities. The use of well-characterized *in vitro* cell culture systems offers an important preclinical strategy to study mechanisms at the basis of cell response to therapeutic agents, including ionizing radiation, possibly leading to a better understanding of the *in vivo* response to the treatment. In this context, we present an integrated analysis of results obtained in an extensive measurement campaign of radiation effects on Caco-2 cells, derived from human colorectal adenocarcinoma. Cells were exposed to X-rays with doses up to 10 Gy from a radiotherapy accelerator. We measured a variety of endpoints at different post-irradiation times: clonogenic survival after ~ 2 weeks; cell cycle distribution, cell death, frequency of micronucleated cells and atypical mitoses, activation of matrix metalloproteases (MMPs) and of different proteins involved in DNA damage response and cell cycle regulation at earlier time points, up to 48 h post-exposure. Combined techniques of flow cytometry, immunofluorescence microscopy, gelatin zymography and western blotting were used. For selected endpoints, we also addressed the impact of the irradiation protocol, comparing results obtained when cells are plated before irradiation or first-irradiated and then re-plated. Caco-2 resistance to radiation, previously assessed up to 72 h post exposure in terms of cell viability, does not translate into a high clonogenic survival. Survival is not affected by the irradiation protocol, while endpoints measured on a shorter time frame are. Radiation mainly induces a G_2_-phase arrest, confirmed by associated molecular markers. The activation of death pathways is dose- and time-dependent, and correlates with a dose-dependent inhibition of MMPs. Genomic aberrations are also found to be dose-dependent. The phosphorylated forms of several proteins involved in cell cycle regulation increase following exposure; the key regulator FoxM1 appears to be downregulated, also leading to inhibition of MMP-2. A unified molecular model of the chain of events initiated by radiation is proposed to interpret all experimental results.

## Introduction

According to data collected by the International Agency for Research on Cancer [available *via* the Global Cancer Observatory platform (1)], colorectal cancer (CRC) is the third most common cancer worldwide in terms of incidence, with a burden of 10.2% of the total ~18.1 million new cancer cases (both sexes, all ages) registered in 2018. When it comes to mortality, CRC is ranked second (after lung cancer), with a burden of 9.2% of the estimated ~9.5 million deaths in the same year. The choice of first-line treatment for CRC patients currently involves a multimodal approach that allows classifying patients in risk groups. This is done considering: tumor-related characteristics, as the presence of metastases (number and localization), stage of tumor progression, possible biochemical markers, etc.; and patient-related factors, such as co-morbidity, prognosis, etc. (2). Based on the risk group, different therapeutic strategies can be adopted. Radiotherapy was originally introduced in CRC treatment as an option to face relapses or oligometastatic states, and has now been established as an essential part of perioperative care. Limitations exist for the application to colon cancer: the colon is mobile (hence the target can be poorly defined), and surrounded by dose-limiting structures (small bowel, kidney and liver). The anatomical structure of the rectum, and the fact that it is situated below the organs that have a limited tolerance to radiotherapy, better justifies the use of radiotherapy for rectal cancer (3). Generally, CRC patients can be treated with radiotherapy and chemotherapy before surgery (neo-adjuvant therapy) or following surgery (adjuvant therapy). Different complementary strategies for neo-adjuvant therapies exist, in particular: a short-course radiotherapy with a 5 × 5 Gy scheme, or a long-course radiotherapy with normofractionated irradiation, for a total dose between 45 and 50.4 Gy, with simultaneous application of chemotherapy (4). Chemotherapy remains the most important adjuvant treatment for colon cancer, while postoperative radiation is currently administered to high-risk patients with rectal cancer. Finally, radiotherapy can also be used for palliation of symptoms, particularly for colon cancer, either from primary lesions, or caused by distant metastases (3).

Overall, radiotherapeutic concepts are recognized as crucial for the primary management of locally advanced rectal cancer (4). Despite advances in treatment modalities however, patients’ survival remains poor. This calls for further research efforts to target drug resistance (5), explore new treatments [including immunotherapy applications (6)], as well as to develop novel combinations of treatments, taking advantage of their possible synergy. In this context, preclinical research greatly benefits from the availability of well-characterized *in vitro* cell and/or tissue systems, which allow to study the mechanisms underlying the response to the treatment in controlled laboratory conditions.

The human cell line Caco-2 has been originally derived from a colon adenocarcinoma. Caco-2 cells have been widely adopted as a model of the intestinal epithelial barrier, thanks to their ability to differentiate and create a functional polarized monolayer when cultured on a porous membrane (7). With such an experimental model, a great deal of studies has focused on measurements of interaction, uptake and cellular transport of drugs and food components, while Caco-2 response to radiation has been less investigated. However, particularly in comparison to other colorectal cancer cell lines, Caco-2 exhibit peculiar features, among which: their poorly aggressive tumor phenotype allow studying mechanisms at play at an early stage of cancer progression, also using radiation as a probe to gain molecular understanding; their p53^null^ status (8), given the well-recognized role of this gene in altering the responses to cancer therapeutic agents (9), offers the chance to focus on p53-independent pathways that might also play an important role in the treatment response. This suggests further investigations to identify and measure some of the unknowns in Caco-2 response to radiation. Recent works with this cell line have focused on its response to different doses of X-rays from a conventional radiotherapy accelerator, with Caco-2 cells alone or co-cultured with peripheral blood mononuclear cells (PBMCs) from healthy donors (10, 11). Doses up to 10 Gy have shown not to alter significantly Caco-2 viability (MTT assay) in a timeframe of 72 h from the irradiation. Also, the epithelial layer integrity (assessed with TEER measurements) seemed not to be affected by radiation only, but permeability was altered and the signaling protein spectrum was modulated when in presence of PBMCs (10). Different questions arose from these results, in particular: i) whether Caco-2 radioresistance in terms of viability, measured in a short time frame, actually translates into a high survival probability when evaluating their clonogenic potential; ii) which mechanisms are at the basis of such radioresistance. A colony formation assay can be used to address the first question. Such assay represents the method of choice to determine cell reproductive death following exposure to radiation, as well as to explore the effectiveness of other agents and their combination, when mimicking a treatment to cancer cells (12). Two essentially different ways exist to perform studies with this assay: in one option, cells are harvested from a stock culture and plated at appropriate density before the treatment; in the second one, cells are first treated and then re-plated, either immediately or with some delay. Treated cells are then followed in time for a sufficient number of replications, leading to colony formation. For a cell line to be fully characterized in terms of radiation response, comparing results obtained with the two options is desirable, as the choice of the protocol can influence cell survival. Limited to the shorter-term effects, a variety of mechanisms driving radiation response can be investigated with different techniques. Flow cytometry is an excellent tool to characterize how the progression in the cell cycle is perturbed by radiation for viable cells, as well as to quantify cell death and identify death mechanisms. As known, activation of cell cycle checkpoints with resulting delays in cell cycle progression might allow cells to successfully repair radiation-induced DNA damage, thus contributing to radioresistance. At the same time though, cells might be forced to exit the cycle (cell death) if the repair is unsuccessful, or might progress fixing alterations leading to genomic aberrations. Complementary information on cell fate in terms of replicative potential can be obtained from morphological features: using fluorescence microscopy, we can monitor cells in their mitotic stage, targeting the occurrence of atypical mitosis, as well as the emergence of micronuclei, as signature of replicative stress and possible markers of chromosomal instability (13).

In the background of the above information and building on already acquired data on the same cell line, we present in this work an integrated analysis of the response of Caco-2 cells to X-ray doses up to 10 Gy. We assessed cell survival with the colony forming assay and we measured a variety of radiobiological endpoints (cell cycle distribution, cell death, micronuclei and atypical mitosis as markers of mitotic instability), obtaining time-series data in the course of 48 h post-irradiation, to make the bridge between long-term replicative potential and short-term mechanisms activated by radiation exposure. For selected endpoints, we also addressed the impact of the irradiation protocol, comparing results obtained when cells are plated before irradiation (here referred to as the “Seed + Treat” method) or first irradiated and then re-plated (referred to as the “Treat + Seed” method). Choosing the most appropriate protocol, we also performed western blotting and gelatin zymography analyses to gain a molecular insight on our dataset, measuring the regulation of different proteins involved in radiation response and of matrix metalloproteases (MMPs).

## Materials and Methods

### Cell Culture and Irradiation Protocols

**Figure 1:**
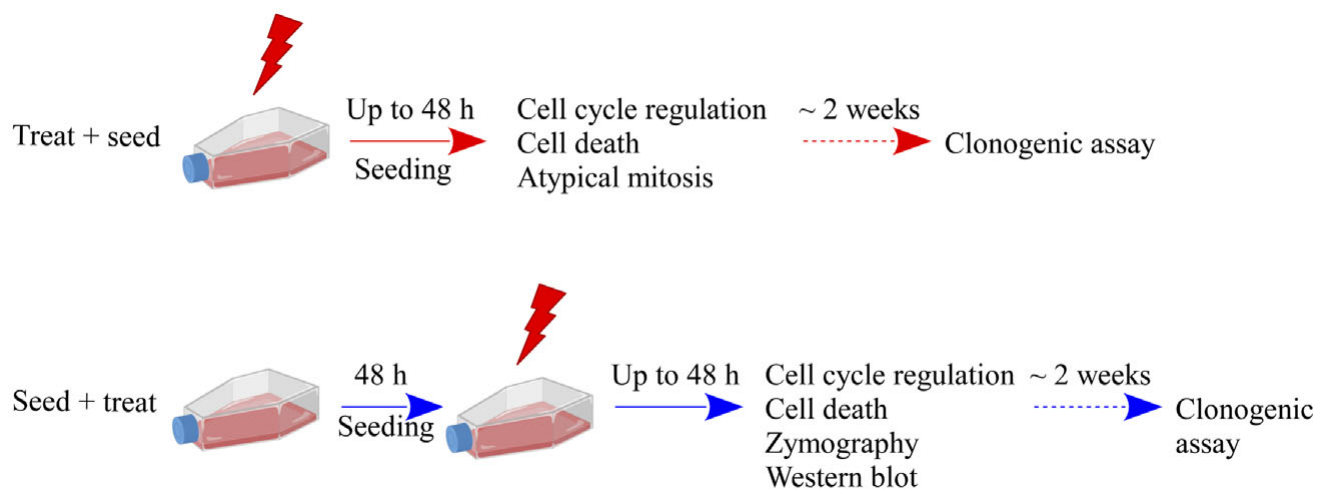
Schematic illustration of the experimental protocols and summary of investigated endpoints. Results presented in this work are obtained with two different experimental protocols: in the “Treat + Seed” protocol cells are first irradiated and then immediately re-plated; in the “Seed + Treat” protocol cells are plated ~48 h before irradiation. In both protocols, cells are exposed to X-rays (doses: sham irradiation at 0 Gy, 2, 5 and 10 Gy) and several endpoints are measured at different time points (6, 24 and 48 h) from the exposure. Cell clonogenic potential is assessed at ~ 14 days from the exposure. Part of illustration created with BioRender.com.

Caco-2 cells were cultured in Dulbecco’s modified Eagle’s medium (DMEM [Gibco]) supplemented with 10% fetal bovine serum (FBS [Life Technologies-Gibco]), 2 mM l-glutamine (Life Technologies-Gibco), 100 U/ml penicillin, 100 μg/ml streptomycin (Life Technologies-Gibco) at 37 °C in a humified atmosphere with 5% CO_2_. Caco-2 cells were at passage 20th to 30th for all experiments. Irradiations were performed at the radiotherapy department of *Istituto di Ricovero e Cura a Carattere Scientifico* (IRCCS) S. Maugeri (Pavia, Italy) with a linear accelerator routinely used for radiotherapy treatment, as previously described (10), with X-ray doses of 0 Gy (control condition, sham), 2, 5, and 10 Gy. Experiments were carried out in parallel with two different experimental protocols, as shown in **Figure 1**, that illustrates the temporal sequence of cell seeding and irradiation (“Treat + Seed” or “Seed + Treat”) and summarizes all measured endpoints. Stock cultures, either for irradiation (“Treat + Seed”) or for further seeding before treatment (“Seed + Treat”) were always at ~70% confluence level.

### Colony Formation Assay

The clonogenic survival of Caco-2 cells was evaluated for both protocols of **Figure 1**. In the “Seed + Treat” protocol, cells were plated at low density (250 cells for the untreated condition and 500 cells for the samples that were later irradiated). In the “Treat + Seed” protocol, cells were exposed to X-rays and, after 30 min, plated at low density (500 cells); also in this case, 250 cells were plated as control for the untreated condition. After 2 weeks from the treatment, colonies were fixed and stained with a solution containing 1% Crystal Violet (Sigma-Aldrich). The day after, colonies were counted by a colony counter (SC6Plus, Stuart). For the highest 10 Gy dose, dedicated replicates were foreseen to verify that seeding 5000 cells does not have an impact on the number of scored colonies. The number of colonies scored for the sham condition defines the plating efficiency. Surviving fraction (SF) data were obtained from colony scoring after normalization to the number of colonies counted for the sham. SF data as a function of dose (D) were fitted with the linear quadratic model to obtain α and β parameters.

### Flow Cytometry Analysis

The cell cycle analysis was performed for both protocols of **Figure 1**. Results were obtained for the distribution of Caco-2 cells (3 × 10^5^ cells in 60 × 15 mm^2^ Petri dishes) in the different cell cycle phases, up to 48 h after X-ray exposure. After irradiation, cells were incubated with 2 µg/µl EdU for 1 h, then fixed following the manufacturer’s instructions with minor changes. Briefly, cells were harvested and fixed 5 min in paraformaldehyde (PF) 4%, then permeabilized in 70% EtOH diluted in physiological buffer (NaCl 0,9% in ddH_2_O). Cells were incubated in a blocking solution (BS) containing 1% BSA (Sigma) in 0,2% PBTween-20 (PBT) for 30 min, then incubated with the primary antibody anti-phospho H3 (Ser 10) [dilution 1:5000, Millipore (RRID : AB_310177)] for 1 h at RT and the secondary antibody goat anti-rabbit IgG 555 [dilution 1:200, Molecular Probes (RRID : AB_141784)] for 30 min at RT. EdU detection was revealed by Click-iT Plus EdU Alexa Fluor 488 Flow Cytometry Assay Kit (Invitrogen, USA) and the DNA content was measured by FxCycle Violet dye (4’,6-diamidino-2-phenylindole, dihydrochloride, Invitrogen).

The cell death analysis was performed for both protocols of **Figure 1**: 3 × 10^5^ Caco-2 cells were seeded in 60 × 15 mm^2^ Petri dishes, and samples collected up to 48 h after X-ray exposure. The analysis to identify apoptosis and necrotic fragments was performed following the manufacturer’s instructions for the eBioscience Annexin V Apoptosis Detection Kit (Invitrogen). All analyses were performed with Attune NxT software v 3.1.

### Cytological Analysis

For the “Treat + Seed” protocol, Caco-2 cells (1.3 × 10^5^) were seeded on coverslips, and their cytological features were evaluated at 48 h after X-ray exposure. Cells were incubated 10 min with a hypotonic solution (75 mM KCl), fixed with 25% glacial acetic acid in methanol and 1% glacial acetic acid in methanol and stained by May-Grünwald Giemsa solutions (14); images were acquired by a Nikon Eclipse i80 microscope.

### Atypical Mitosis and Micronuclei

For the “Treat + Seed” protocol, the occurrence of atypical mitosis and micronucleated cells was quantified with fluorescence microscopy. Caco-2 cells (1.8 × 10^5^) were seeded on coverslips and cultured for 48 h, then fixed in 4% PF and permeabilized in 70% EtOH diluted in ddH_2_O. Coverslips were incubated with BS for 30 min at RT, then with the primary antibody anti-phospho H3 (Ser 10) [dilution 1:5000, Millipore (RRID : AB_310177)] for 1 h at RT and the secondary antibody goat anti-rabbit IgG 488 [dilution 1:100, Molecular Probes (RRID: AB_1904025)] for 30 min at RT, finally washed with Hoechst 33342 dye (Abnova) and mounted with Mowiol (Calbiochem) containing 0.25% 1,4-diazabicyclo-octane (Sigma-Aldrich) as antifading agent. Mitotic spindle and micronuclei were visualized with fluorescent microscopy (Olympus BX51). Images were acquired by digital CCD camera (Retiga-2000R). Scoring was performed manually.

### Gelatin Zymography

For the “Seed + Treat” protocol, measurements of Matrix Metalloproteases (MMP-9 and MMP-2) in the culture medium were performed following the experimental procedure already published in (10), with minor changes. Conditioned media (500 μl, from samples used for Western Blotting analysis, see later) were collected, centrifuged at 4,600*g* (Thermo Scientific CL31R) and supernatants mixed in Sample Buffer 2× (0.5 M Tris-HCl pH 6.8, 20% glycerol, 10% SDS, 0.1% Bromophenol blue), ratio 1:1, and stored at −80°C. 20 μl of each sample were loaded on a 10% polyacrylamide gel containing 1 mg/ml Bovine Type B Gelatin (Sigma-Aldrich). Gels were stained with Coomassie Blue R-250 (0.5% w/v) and subsequently de-stained and acquired with Image Gel Analyzer (Bio-Rad) (15).

### Western Blotting

Caco-2 cells (3 × 10^5^ seeded, following the “Seed + Treat” protocol) were collected by trypsinization after 6, 24 and 48 h after radiation exposure. Cells were centrifuged at 300*g* for 3 min at RT, washed in PBS, centrifuged at 3,400*g* for 5 min at RT and the pellets were stored at −80°C. Pellets were sonicated in a lysis buffer [10 mM Tris-HCl, 2.5 mM MgCl_2_, 0.5% Triton X-100, 1 mM PMSF, 1× Nuclear Extraction Phosphatase Inhibitors (Caymann Chemical Company), 1× Nuclear extraction Protease Inhibitors Cocktail (Caymann Chemical Company) and 25 U/µl Benzonase^®^] at 50% (Omni Sonic Ruptor 400) for 10 seconds and incubated for 20 min at RT. Samples were centrifuged at 10,000*g* for 5 min at 4°C, and the supernatants were quantified with Bradford (VWR) method at UV-3100 spectrophotometer (VWR). For each sample, 30 μg of proteins were mixed with a 3× SDS-loading buffer (65 mM Tris-HCl pH 7.4, 100 mM DTT, 10% glycerol, 1% SDS, 0.02% Bromophenol blue). Proteins were electrotransferred to nitrocellulose membranes through a semi-dry system, and membranes were blocked for 30 min in 5% BSA in PBST buffer. Proteins were detected with specific primary antibodies: anti-cdc25C (dilution 1:1000, Cell Signalling [RRID : AB_560956]), anti-P-cdc25C (S216) [dilution 1:1000, Cell Signalling (RRID : AB_331215)], anti-Chk2 [dilution 1:1000, Cell Signalling (RRID : AB_2229490)], anti-P-Chk2 (T68) [dilution 1:1000, Cell Signalling (RRID : AB_331479)], anti-H2A.X [dilution 1:1000, Cell Signalling (RRID : AB_10860771)], anti-P-H2A.X (S139) [dilution 1:1000, Abcam (RRID : AB_1640564)], anti-CyclinB1 [dilution 1:1000, Cell Signalling (RRID : AB_2233956)], anti-FoxM1 [dilution 1:1000, Cell Signalling (RRID : AB_2798842)] and anti-GAPDH [dilution 1:1000, Cell Signalling (RRID : AB_10622025)]; the secondary HRP-conjugated antibodies were used: sheep anti-mouse IgG [dilution 1:2000, GE Healthcare (RRID : AB_772210)] and donkey anti-rabbit IgG [dilution 1:2000, GE Healthcare (RRID : AB_772206)]. To reveal protein levels, a chemiluminescent enhancer (Bio-Rad) was used. Densitometric analysis was performed using ImageJ software (NIH, MD).

### Statistical Analysis

For the different endpoints, each experimental value represents the mean of at least three independent measurements; errors are given as standard error of the mean (SEM) or standard deviation (SD) (details are specified in figure captions). To determine whether radiation exposure and time induced a statistically significant change in experimental results, we performed a two-way analysis of variance (ANOVA) test with post-hoc pairwise t-test for repeated measurements, with Bonferroni correction for data on cell cycle perturbation and cell death. The statistical significance (p) was calculated by means of the two-tailed Student’s t-test for data on mitotic instability markers, MMPs activity and western blot analysis. Details are given in the Figure captions.

## Results

### Cell Survival

**Figure 2:**
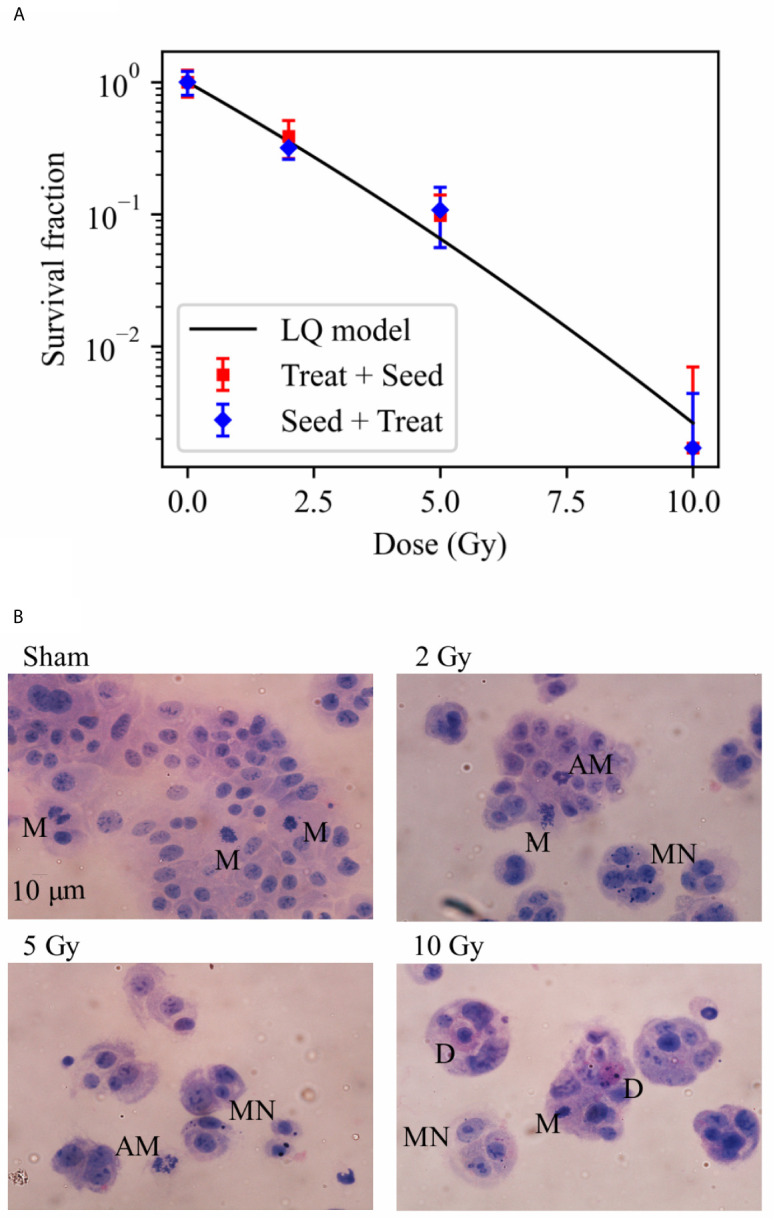
Cell survival and cytological staining. **(A)** Survival fraction (SF) of Caco-2 cells exposed to 0 (sham), 2, 5 and 10 Gy of X-rays following the “Treat + Seed” and “Seed + Treat” experimental protocols (details in the text). Data are mean ± SD, obtained from ≥3 independent experiments. SF data are fitted with the linear quadratic model to obtain α and β parameters, details in the text. **(B)** Illustrative cytological images of cells (scale bar: 10 μm) 48 h after exposure to the same X-ray doses for the “Treat + Seed” protocol, with evidence of micronuclei (MN), typical and atypical mitoses (M and AM) and cell death (D) events.

In **Figure 2A**, we report data on the survival fraction of Caco-2 cells exposed to different doses of X-rays as measured with the two different protocols schematized in **Figure 1**. Clonogenic cell survival decreases with increasing dose, and data are almost the same for the “Seed + Treat” and “Treat + Seed” protocols. In particular, very few colonies can be scored for the 10 Gy irradiation condition, leading to an almost negligible survival fraction. As data for the two protocols are always within statistical uncertainties, a single fit with the linear quadratic model was performed, leading to the following parameters: α = 0.50 ± 0.09 Gy^−1^ and β = 0.01 ± 0.01 Gy^−2^.

Illustrative images of cells (seeded at high density, see Material and Methods), obtained 48 h after the exposure for the “Treat + Seed” protocol (**Figure 2B**) and analyzed in relation to survival data, suggest what follows: in the Sham condition (0 Gy) colonies soon become dense and well distributed; a first alteration of these features is already evident following irradiation with 2 Gy, and it becomes more evident at 5 and 10 Gy: replicating cells form “colonies” that are smaller in dimensions and cell number, and the frequency of cells with morphological features like micronuclei (MN) and atypical mitoses (AM) increases, as well the frequency of cell death (D) events. What is observed at 48 h for the 10 Gy condition seems to indicate that cells initially try to cope with the radiation exposure and attempt to replicate and form colonies (later discussed in relation to cell viability at the same dose from previous measurements), though numerical data from **Figure 2A** demonstrate that cell death is prevailing in the longer term.

### Cell Cycle Perturbations

**Figure 3:**
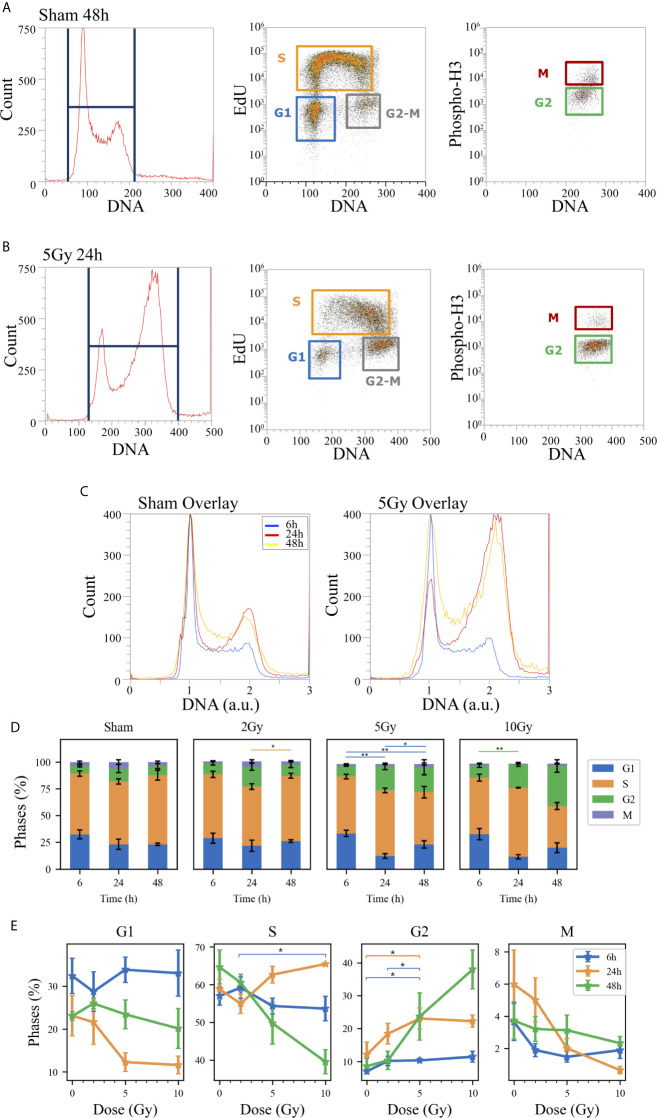
Cell cycle analysis with flow cytometry following the “Treat + Seed” protocol. Hierarchical gating strategy for the distribution of Caco-2 cells in the cell cycle demonstrated with illustrative data for: **(A)** Sham condition at 48 h; **(B)** 5-Gy-irradiated cells at 24 h. From left to right in both panels: monoparametric distribution of FxCycle violet signal with gate on all cells; biparametric plot of EdU vs. FxCycle signals for all cells with gates on G_1_, S and G_2_/M phases; biparametric plot of Phospho-H3 vs. FxCycle signals for all G_2_/M cells with gates on G_2_ and M phases. **(C)** Illustrative relative DNA content distributions for the whole cell population (average FxCycle signal for cells in G1 normalized to 1) for the sham (left) and 5-Gy condition (right) overlayed at different time points. **(D)** Percentages of cells in G_1_, S, G_2_ and M for the different irradiation conditions as a function of time. **(E)** Percentages of cells in each phase for the different time points as a function of X-ray dose (same data as in panel D, lines are a guide to the eye). Data are mean ± SEM, obtained from ≥3 independent experiments. Statistical significance (post-hoc pairwise comparisons with Bonferroni correction) is as follows: *p < 0.05, **p < 0.01.

**Figure 4:**
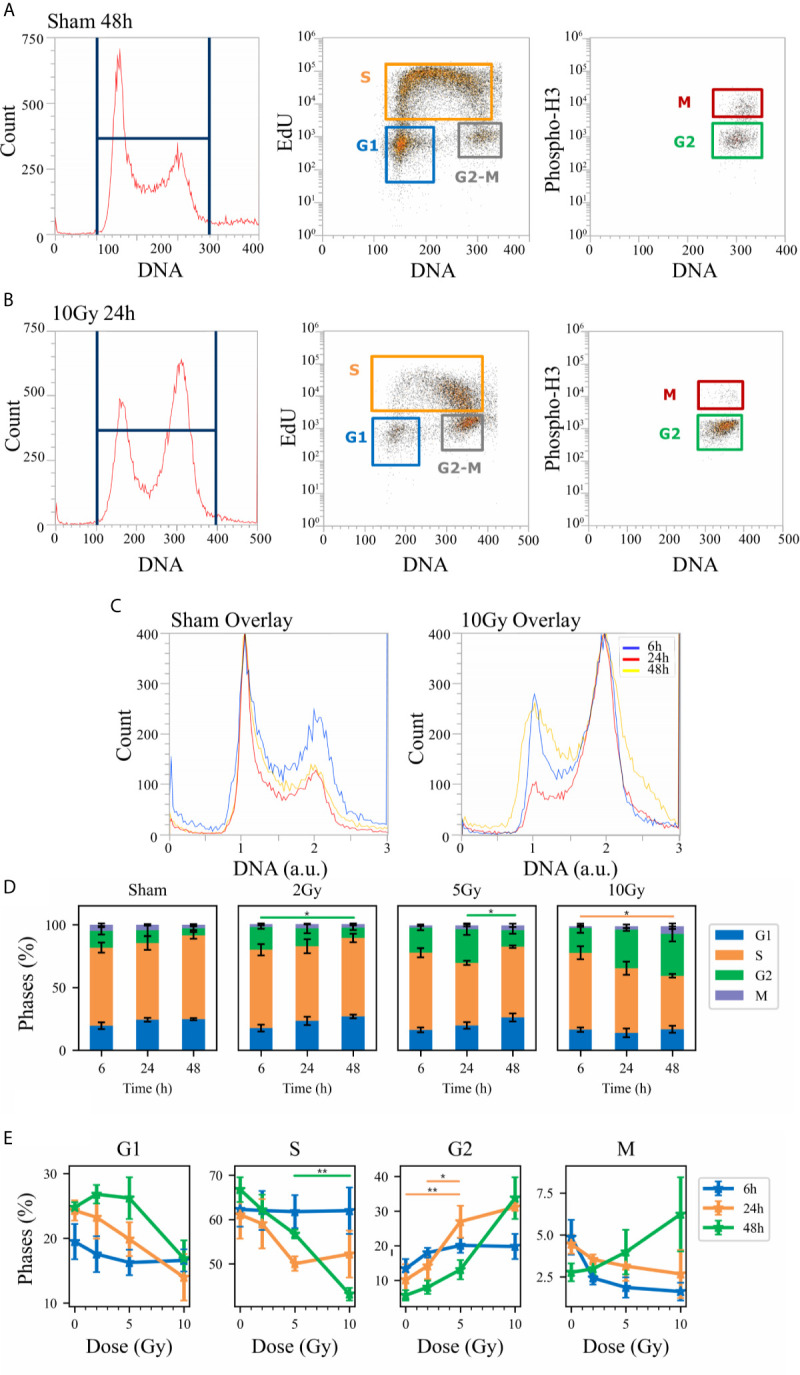
Cell cycle analysis with flow cytometry following the “Seed + Treat” protocol. Hierarchical gating strategy for the distribution of Caco-2 cells in the cell cycle demonstrated with illustrative data for: **(A)** Sham condition at 48 h; **(B)** 10-Gy-irradiated cells at 24 h. From left to right in both panels: monoparametric distribution of FxCycle violet signal with gate on all cells; biparametric plot of EdU vs. FxCycle signals for all cells with gates on G_1_, S and G_2_/M phases; biparametric plot of Phospho-H3 vs. FxCycle signals for all G_2_/M cells with gates on G_2_ and M phases. **(C)** Illustrative relative DNA content distributions for the whole cell population (average FxCycle signal for cells in G1 normalized to 1) for the sham (left) and 10-Gy condition (right) overlayed at different time points. **(D)** Percentages of cells in G_1_, S, G_2_ and M for the different irradiation conditions as a function of time. **(E)** Percentages of cells in each phase for the different time points as a function of X-ray dose (same data as in panel D, lines are a guide to the eye). Data are mean ± SEM, obtained from ≥3 independent experiments. Statistical significance (post-hoc pairwise comparisons with Bonferroni correction) is as follows: *p < 0.05, **p < 0.01.

Flow cytometry analysis was performed to evaluate Caco-2 cell cycle perturbation after exposure to different doses of X-rays. Measurements were performed for both methodological approaches; results are summarized in **Figures 3** and **4**, respectively for the “Treat + Seed” and “Seed + Treat” protocols. For both figures, the structure of the panel is as follows: panel A shows illustrative flow cytometry data for the sham condition at a selected time point (48 h); panel B shows the same set of data for a selected irradiation condition (dose and time point). The first distribution in both panels is obtained with FxCycle violet, a fluorescent stain that marks DNA. The measured fluorescence intensity is proportional to the overall amount of DNA in a cell, and this allows to obtain an overview of how the asynchronous cycling population is distributed in the cell cycle with a single parameter distribution. In the central plot, the signal from the S-phase specific marker EdU is correlated to the FxCycle signal, thus allowing the gating of cells in G_1_ (low DNA amount, no EdU), cells in G_2_-M (double DNA amount, no EdU) and cells in the S-phase, that are replicating DNA (increasing FxCycle signal and positive EdU signal). In the last plot, cells in G_2_-M are further analyzed looking at the correlation between the M-phase specific marker anti-phosphoH3(Ser10) and the FxCycle signal, thus allowing the gating of cells in the M and G_2_-phase separately. Panels A and B therefore demonstrate the gating strategy that is applied to obtain quantitative data on the percentage of cells in each phase, normalizing cell counts in the gate for a specific phase to the sum of counts for all four phases. Panel C further shows for illustrative purposes relative DNA content distributions for the whole cell population, obtained normalizing to 1 the average of the FxCycle signal for cells in G_1_. Such normalized distributions are shown for different time points for the sham condition (left) and for a selected irradiation condition (right). Histograms in panel D finally report the full dataset of percentages of cells in the different cell cycle phases as a function of time, and for all the irradiation conditions. Scatter plots in panel E show how the percentage of cells in a specific phase at the different time points after irradiation varies as a function of the X-ray dose.

When comparing data (see **Figures 3D** and **4D**) it is important to keep in mind that the difference in the protocols implies a sort of an overall “time-shift” in the progression of cells in the cycle already for the non-irradiated condition: indeed, the 48 h condition for the “Treat + Seed” protocol (**Figure 3D**) resembles the 6 h condition for the “Seed + Treat” protocol (**Figure 4D**). Following the “Treat + Seed” protocol, cells are first treated and then seeded, and soon after seeding they start to progress in the cycle. Following the “Seed + Treat” protocol, at the moment of being irradiated cells have already been progressing in the cycle starting from the initial seeding: therefore, at early time points after irradiation, cells are found in a condition that is similar to that reached at later time points for the “Treat + Seed” protocol.

Looking at **Figures 3E** and **4E**, the perturbation of the cell cycle distribution is more clearly assessed as a function of radiation dose: in particular, an increase of cells in G_2_ is observed after exposure, which suggests a possible activation of the G_2_-M checkpoint. For the “Treat + Seed” protocol (**Figure 3E**), the percentage of cells in G_2_ is higher at 24 h for doses below 5 Gy, while at 10 Gy the block seems to be more persistent in time and almost 40% of the total cell population is found in G_2_ at 48 h after the exposure. For the “Seed + Treat” protocol (**Figure 4E**), the percentage of cells in G_2_ seems to be higher at 24 h for all exposure conditions, though larger error bars are visible in the scatter plot. In both cases, the increase of cells in G_2_ seems to happen mainly at the expense of the S-phase, which is the more populated already in the sham condition (with percentages around ~60% at all time points). A dose-dependent decrease of cells in G_1_ is also observed at 24 h for both the “Treat + Seed” and “Seed + Treat” protocols. A decreasing trend for cells in M can also be guessed as radiation dose increases, but the percentages of mitotic cells are always small and with too large error bars to make conclusions.

### Cell Death

**Figure 5:**
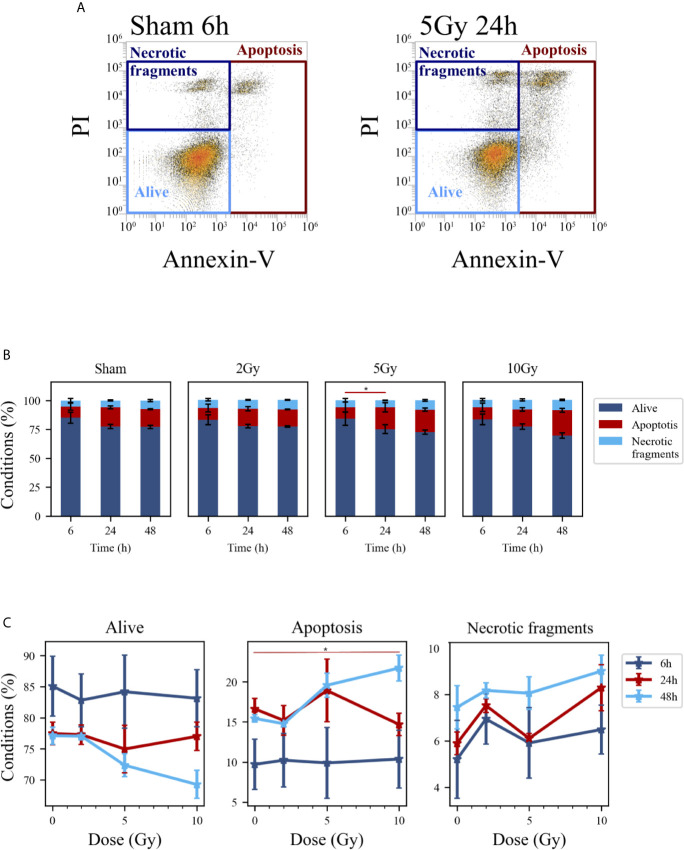
Cell death analysis with flow cytometry following the “Treat + Seed” protocol. **(A)** Gating strategy to identify in the Caco-2 population living cells (Alive), apoptotic cells (Apoptosis) and any intermediate cell death form (Necrotic fragments) in the biparametric plot of Annexin V vs. PI signals, demonstrated with illustrative data for: sham condition at 6 h (left); 5-Gy-irradiated cells at 24 h (right). **(B)** Percentages of living, apoptotic cells and necrotic fragments for the different irradiation conditions as a function of time. **(C)** Percentages of living, apoptotic cells and necrotic fragments for the different time points as a function of X-ray dose (same data as in panel B, lines are a guide to the eye). Data reported are mean ± SEM, obtained from ≥3 independent experiments. Statistical significance (post-hoc pairwise comparisons with Bonferroni correction) is as follows: *p < 0.05.

**Figure 6:**
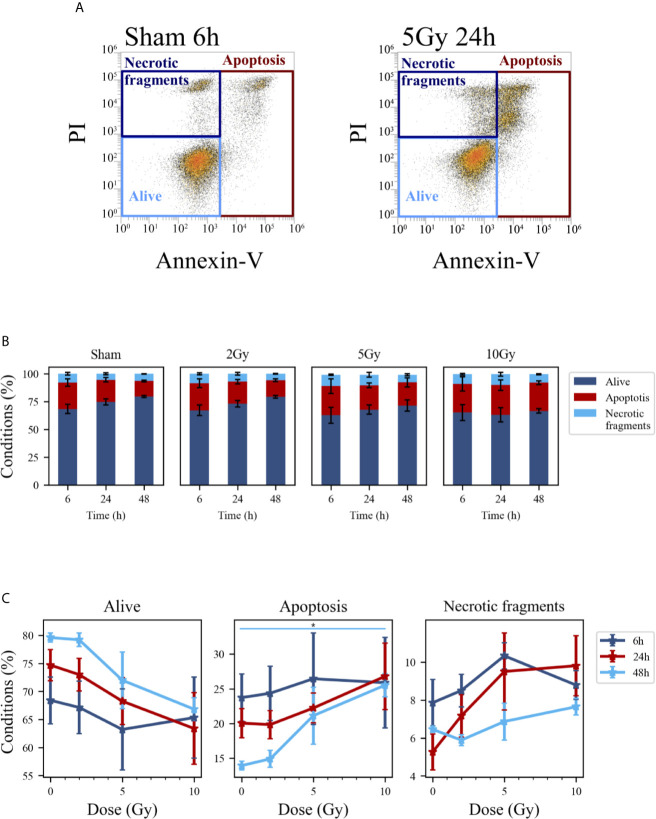
Cell death analysis with flow cytometry following the “Seed + Treat” protocol. **(A)** Gating strategy to identify in the Caco-2 population living cells (Alive), apoptotic cells (Apoptosis) and any intermediate cell death form (Necrotic fragments) in the biparametric plot of Annexin V vs. PI signals, demonstrated with illustrative data for: sham condition at 6 h (left); 5-Gy-irradiated cells at 24 h (right). **(B)** Percentages of living, apoptotic cells and necrotic fragments for the different irradiation conditions as a function of time. **(C)** Percentages of living, apoptotic cells and necrotic fragments for the different time points as a function of X-ray dose (same data as in panel B, lines are a guide to the eye). Data are mean ± SEM, obtained from ≥3 independent experiments. Statistical significance (post-hoc pairwise comparisons with Bonferroni correction) is as follows: *p < 0.05.

Cell death events were analyzed and quantified by flow cytometry with the Annexin V/PI method for both methodological approaches; results are summarized in **Figures 5** and **6**, respectively for the “Treat + Seed” and “Seed + Treat” protocols. For both figures, panels have the following structure: panel A shows illustrative flow cytometry data for the earliest time point (6 h) of the sham condition (left) and for a selected irradiation condition (5 Gy, 24 h). Panel A therefore demonstrates the gating strategy in the biparametric plot: cells that are negative for both signals (-/-) are identified as living cells (label: “Alive”); cells positive for Annexin V and negative for PI (+/-) are identified as apoptotic cells (“Apoptosis”); finally, events with double positive signals, Annexin V and PI (+/+), are identified as due to “Necrotic fragments” (or intermediate cell death forms). Histograms in panel B report the full dataset of percentages of cells in the different classes as a function of time, and for all the irradiation conditions (normalization is to the sum of events in the three gates). Scatter plots in panel C show how the percentage of living, apoptotic and necrotic cells at the different time points after irradiation varies as a function of the X-ray dose.

At the earliest time point for the sham conditions, living cells represent respectively ~85% and ~68% of the whole population for the “Treat + Seed” (**Figure 5B**) and “Seed + Treat” (**Figure 6B**) protocols. As noted for cell cycle data however, considering the “time-shift” that is caused by the differences in the two protocols, the percentage of living cells for the “Treat + Seed” method at 48 h gets closer to that for the “Seed + Treat” method at 6 h. As a function of radiation dose, the percentage of living cells decreases in favor of apoptotic cells for the “Treat + Seed” method at 48 h (**Figure 5C**), which starts to be visible at 5 Gy, becoming more evident following exposure to the highest 10 Gy dose. For the “Seed + Treat” method (**Figure 6C**) a dose-dependent increase in the percentage of apoptotic cells can be observed already at 24 h, and the effect is more marked at 48 h, also starting from a lower percentage of apoptotic cells in the sham condition. The percentage of necrotic fragments seems also to increase as a function of radiation dose at 24 h, though data are affected by large statistical variations.

### Genomic Aberrations

**Figure 7:**
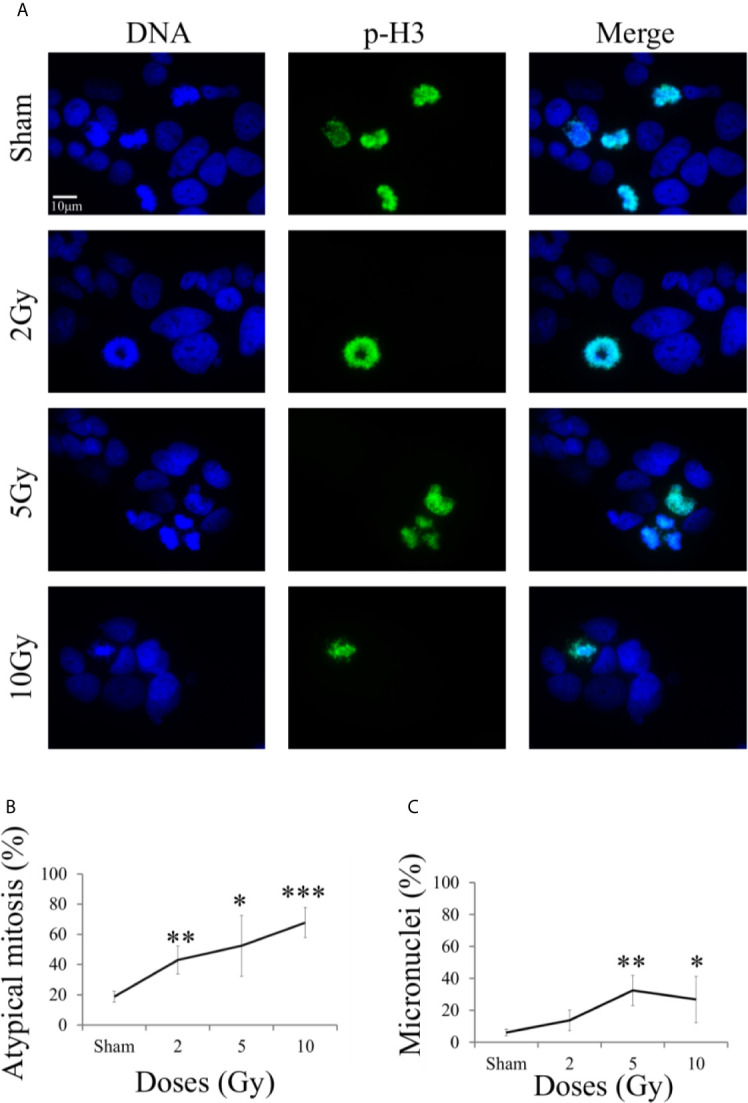
Mitotic instability markers with fluorescence microscopy. For Caco-2 cells 48 h after X-ray exposure following the “Treat + Seed” protocol: **(A)** Illustrative fluorescence microscopy images (scale bar: 10 μm) for the different irradiation conditions, obtained with Hoechst (for nuclear DNA, left) and pH3 (as a specific mitotic marker, center) staining and their merge (right), used to identify micronucleated cells and cells presenting mitotic atypia (*e.g.* anaphase bridges, multipolar, ring, dispersed, asymmetrical, lag-type mitoses); **(B)** Percentage of atypical mitoses (normalized to all analyzed mitotic cells) as a function of X-ray dose (line is a guide to the eye); **(C)** Percentage of micronucleated cells (normalized to all analyzed cells) as a function of X-ray dose (line is a guide to the eye). Data are mean ± SD, obtained from ≥3 independent experiments. Statistical significance (Student’s t-test) is as follows: *p < 0.05, **p < 0.01, ***p < 0.001.

Micronucleated cells and cells presenting mitotic atypia (*e.g.* anaphase bridges, multipolar, ring, dispersed, asymmetrical, lag-type mitoses) were identified as markers of mitotic instability following exposure to X-rays. The morphological analysis was carried out 48 h after exposure following the “Treat + Seed” protocol, using fluorescence microscopy images (**Figure 7A**) obtained with DNA staining (left column) and pH3(Ser10) antibody (central column), a specific mitotic marker (merged images are shown in the right column).

On average, mitotic events were about 10% of all analyzed cells already in the sham condition, including both typical and atypical mitosis, with a 1,8% of atypical mitosis. **Figure 7B** shows the percentage of atypical mitoses (normalized to all mitotic cells) as a function of radiation dose. The percentage of atypical mitosis increases as a function of dose in a seemingly linear way, starting from ~20% in the sham condition and reaching more than 60% at 10 Gy. The high percentage of atypical mitoses already for non-irradiated cells can be seen as characteristic of a tumor cell line.

A similar trend was observable also for micronuclei formation (**Figure 7C**, percentages referring to the total number of analyzed cell), caused by an incorrect chromosomal segregation during mitosis. However, the percentage of micronucleated cells reaches its maximum (~37%) at 5 Gy. The further decrease observed at 10 Gy can be attributed to difficulties in the identification of micronucleated cells, due to the concomitant increase in the number of cells that have activated cell death mechanisms at the same 48 h time point (see **Figure 5C**).

### Gelatin Zymography

**Figure 8:**
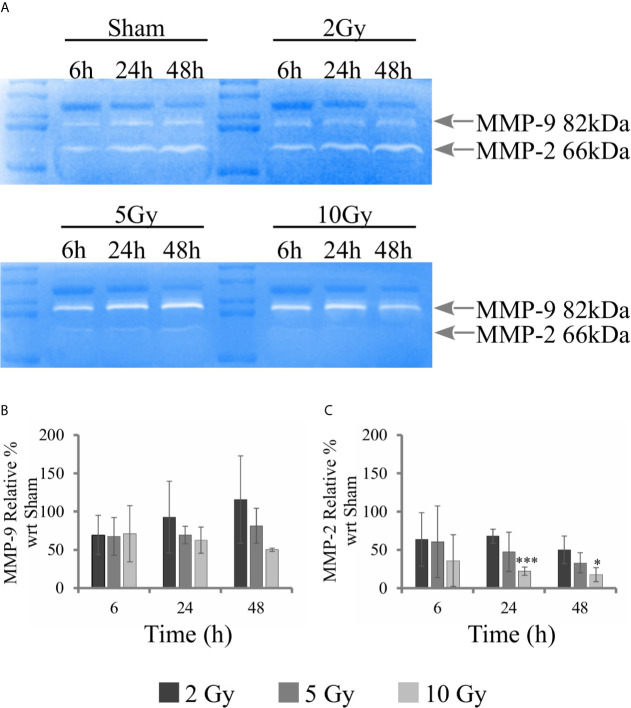
Activity of MMPs with gelatin zymography analysis. For Caco-2 cells following the “Seed + Treat” protocol: **(A)** Representative images of gelatin zymography for the different irradiation conditions and time points, with identification of bands corresponding to MMP-9 and MMP-2; Quantification of MMP activity as a function of time for the different irradiation conditions (normalization is to the sham condition at the same time point) for: **(B)** MMP-9; **(C)** MMP-2. Data reported are mean ± SD, obtained from ≥3 independent experiments. Statistical significance (Student’s t-test) is as follows: *p < 0.05, **p < 0.01, ***p < 0.001.

Gelatin zymography experiments were performed to evaluate the activity of metalloproteases MMP-9 and MMP-2. Measurements were performed on conditioned media collected from samples of the “Seed + Treat” protocol. **Figure 8A** shows representative images of gelatin zymography. **Figures 8B, C** reports the quantification of MMP-9 and MMP-2 activity, respectively: after quantification of the intensity of white bands, data are expressed as relative percentage to the sham condition at the same time point. Overall, MMP-9 activity seems not to be affected by radiation. A decreasing trend as a function of dose could be guessed for the latest 48 h time points, but statistical variations are too high to make any conclusive statement. MMP-2 activity is inhibited by radiation, and the effect is visible both at 24 h and 48 h, being statistically significant for the highest 10 Gy dose.

### Western Blotting

**Figure 9:**
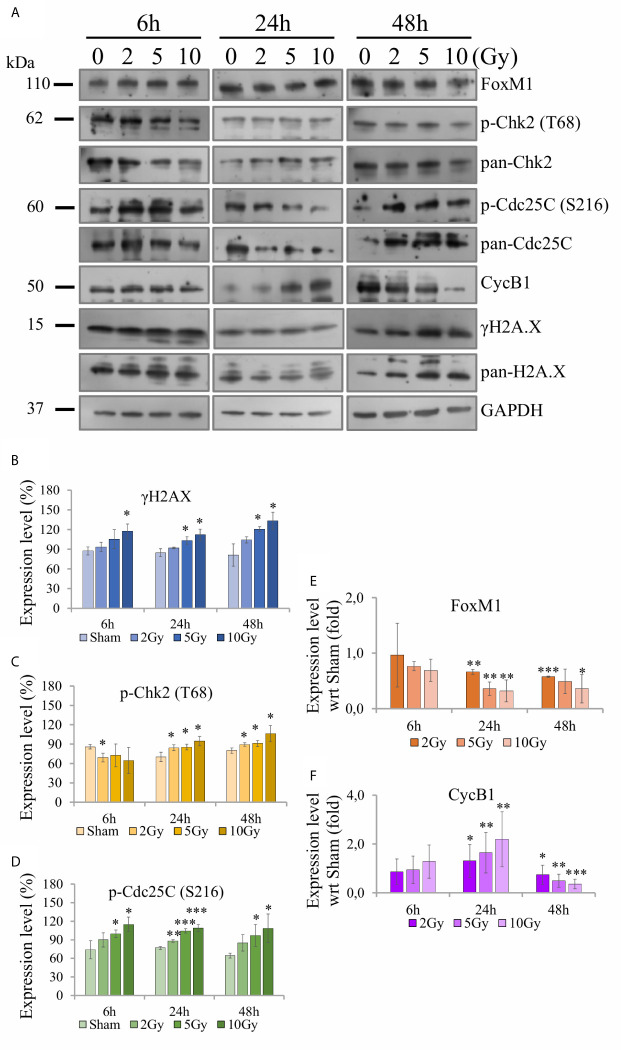
Activity of proteins involved in DNA damage and cell cycle regulation with Western blot analysis. For Caco-2 cells following the “Seed + Treat” protocol: **(A)** Illustrative images of films with expression patterns of all measured proteins at the different time points and irradiation conditions. Selected proteins are quantified and expressed as a function of time for the different irradiation conditions as follows: **(B)** ratio γ-H2AX (S139) to total H2AX; **(C)** ratio phospho-Chk2 (T68) to total Chk2; **(D)** ratio phospho-Cdc25C (S216) to total Cdc25C; **(E)** ratio FoxM1 to the GAPDH loading control, normalized to the same ratio for the sham condition at the same time point; **(F)** ratio CycB1 to the GAPDH loading control, normalized to the same ratio for the sham condition at the same time point. Data reported are mean ± SD, obtained from ≥3 independent experiments. Statistical significance (Student’s t-test) is as follows: *p < 0.05, **p < 0.01, ***p < 0.001.

Western blotting analysis was performed on cell samples obtained following the “Seed + Treat” protocol, to offer a molecular interpretation of the collected dataset. We evaluated the regulation of several proteins involved in the radiation response, including markers of DNA damage (γ-H2AX) and a variety of proteins more specifically involved in cell cycle progression, focusing on the G_2_/M transition (Chk2, Cdc25C, CycB1), as well as of the key regulator FoxM1. **Figure 9A** shows for illustration purposes images of films with expression patterns of all measured proteins at the different time points and irradiation conditions (including GAPDH as loading control). Data are quantified and presented as follows in the different panels of **Figure 9**: for H2AX (**Figure 9B**), Chk2 (**Figure 9C**) and Cdc25C (**Figure 9D**) we plot the intensity ratio of their phosphorylated form (respectively, γ-H2AX (S139), phospho-Chk2 (T68), phospho-Cdc25C (S216)) to total protein content for the different time points, as a function of X-ray dose; for CycB1 (**Figure 9F**) and FoxM1 (**Figure 9E**) we plot the intensity ratio of the protein content to the loading control, for the different time points, after further normalization to the result for the sham condition at the same time point (bar set to 1, not shown in the histogram).

The γ-H2AX signal is found to increase as a function of dose at all time points (**Figure 9B**). Also signals from phospho-Chk2 (**Figure 9C**) and phospho-Cdc25C (**Figure 9D**) increase in a dose-dependent manner, the effects seeming to be more pronounced at later time points. FoxM1 (**Figure 9E**) is inhibited as dose increases at 24 h and 48 h, while no-significant variation is observed at the earliest time points. Also CycB1 (**Figure 9F**) is not affected at 6 h, its concentration with respect to the sham condition is first increased as a function of dose at 24 h and then found to decrease.

## Discussion

The large data set presented in this work on colorectal adenocarcinoma Caco-2 cells exposed to X-ray doses up to 10 Gy offers the opportunity to conduct an integrated analysis of a variety of endpoints, measured with different techniques, to characterize the radiation response of this cell line, also gaining molecular insight into underlying mechanisms elicited by radiation.

First of all, new data allow assessment of the impact of the experimental protocol, in terms of temporal sequence of cell seeding and irradiation (**Figure 1**), on measured radiobiological endpoints. To this aim, we compared two protocols, both established and in use in different laboratories, whose differences have been particularly discussed for the clonogenic assay (12): i) in the “Treat + Seed” method, cells are first irradiated and then seeded for further measurements (either immediately, as in this study, or introducing a time delay, also to address the issue of sub-lethal damage repair). Generally speaking, this approach seems more commonly adopted in pharmacological studies; ii) in the “Seed + Treat” method on the contrary, cells are first seeded and then irradiated after an appropriate time interval (~ 48 h in our study, to allow for a whole cell cycle duration after seeding), which is more common practice for radiobiological studies. In both cases, cell samples are further analyzed at the desired time point after irradiation. Our results (**Figure 2**) indicate that Caco-2 clonogenic potential, measured in terms of cell colonies scored after 2 weeks from the irradiation, is not affected by the choice of the protocol. Results for endpoints measured at earlier time points (up to 48 h from the irradiation) are instead found to be different between the two protocols, also for the non-irradiated condition. This has been observed for the distribution of cells in cell cycle phases (**Figures 3** and **4**) and for cell death events (**Figures 5** and **6**) measured by means of flow cytometry. As already noted in the description of results, differences observed for the sham condition can be mainly attributed to an overall “time-shift” between cell populations that is induced by differences in the two protocols. Starting from an asynchronous and proliferating cell population, with a basal percentage of dead cells, unirradiated cells at the earlier time point for the “Seed + Treat” protocol (temporal sequence: seeding, ~ 48-h interval, sham-irradiation and then early measurement) are found to be in a similar condition with respect to unirradiated cells at the latest time point for the “Treat + Seed” protocol (temporal sequence: sham-irradiation, ~ immediate seeding, measurement at ~ 48 h). This needs to be taken into account when comparing results obtained with the two methods also for the irradiated conditions, in which radiation acts as a perturbation of cell populations differently progressing in time. It is also interesting to notice that statistical variations associated with measurements following the “Seed + Treat” protocols generally appear to be higher. Nevertheless, similar conclusions can be drawn in terms of radiation effects on cell cycle and cell death, particularly in terms of the accumulation of cells in G_2_ following irradiation and of the dose-dependent increase in the percentage of apoptotic cells. Quantitative results and the specific perturbation pattern (*e.g*. the time point at which the maximum effect is reached) remain dependent on the chosen protocol. Most importantly, measured differences in the initial response up to 48 h have no consequence on long-term cell replicative potential, which can be equally assessed with either of the two methods. For the additional endpoints measured in this work, we limited ourselves to the most appropriate protocol, depending on the specific endpoint under consideration. As an example, we have previously shown that the measurement of MMPs activity can be significantly altered if cells are seeded after treatment, as MMPs are activated by the use of trypsin, which is used to detach cells after irradiation in the “Treat + Seed” method (15). As a consequence, MMPs activation induced by trypsin can overcome any radiation-induced inhibition effect (later discussed), thus leading to wrong conclusions on the role of MMPs in the radiation response.

Results on Caco-2 cell survival, complemented by cytological staining (**Figure 1**), fluorescence microscopy images (**Figure 7**) and quantification of cell death (**Figure 5**), further add to previous findings (10, 11) that led to the description of this cell line as “radioresistant”: in particular, in our previous works, Caco-2 viability measured with the MTT assay was found to remain as high as in the sham condition for cells irradiated with 10 Gy and followed in time up to 72 h from the exposure. The percentage of dead cells measured with the Trypan Blue assay was found to increase in a dose-dependent manner, starting from a basal condition at ~10% and reaching a maximum at around 20% at 24 h and 48 h. Results presented in this work indicate that such behavior, that can be described as “radioresistant” in terms of short-term effects, does not translate into a persistent clonogenic potential: already at 2 Gy, only ~30% of cells are able to form colonies at ~2 weeks, this survival probability decreases to ~10% at 5 Gy and few or no colonies are observed at 10 Gy. Results at 5 Gy are quite in agreement with what observed in a previous work after X-ray exposure of Caco-2 cells seeded 48 h before irradiation (16), while a slightly higher surviving fraction was assessed by the authors at 2 Gy (~ 50%). In this latter work, colonies were scored at 11 instead of 14 days, and the number of cells seeded, used for calculating the plating efficiency, was determined with a separate sample that was fixed immediately after cells were allowed to adhere, which could lead to a higher plating efficiency and higher surviving fraction data. Caco-2 exposure with a ^137^Cs γ-ray source (cells seeded 12 h before) led to the same survival fraction at 14 days at 2 Gy (~29%), but to a lower survival (~2%) at 5 Gy in (17), with α = 0.62 ± 0.05 Gy^−1^ and β = 0.03 ± 0.03 Gy^−2^ for the linear quadratic model, quite in agreement with our parameter estimates. Data in (18) for Caco-2 (seeded 18 h prior to irradiation), also exposed to ^137^Cs γ-ray source, show instead higher survival fractions (around 20 - 30% at 5 Gy and a few % at 10 Gy) at 8 - 14 days, with α = 0.09 Gy^−1^ and β = 0.01 Gy^−2^ (parameter uncertainties not given in the text), at odds with our findings. In the context of this latter work, Caco-2 cells are described as radioresistant in terms of clonogenic potential, also compared to other colorectal cancer cell lines, which is associated with their p53^null^ status. Such variability in experimental results could be (at least partially) attributed to the heterogeneity of Caco-2 cell line and to the impact of cultivation condition, in *e.g.* selecting subpopulations of cells with properties that may differ from the original cell line (7). This further calls for the need of integrating the measurements of different endpoints (as stated above, also considering the impact of specific treatment protocols) to have a well-characterized cell model, which is the approach followed in this work. Images for cytological analysis show that cells, even if irradiated with 10 Gy, initially try to cope with the radiation insult and to replicate within 48 h from the exposure, which might explain the persistent viability measured in our previous works in the same time interval. However, cell death events start occurring with an increased frequency due to radiation, which can be observed both from cytological and fluorescence microscopy images, and quantified with the Annexin V/PI method by flow cytometry.

**Figure 10:**
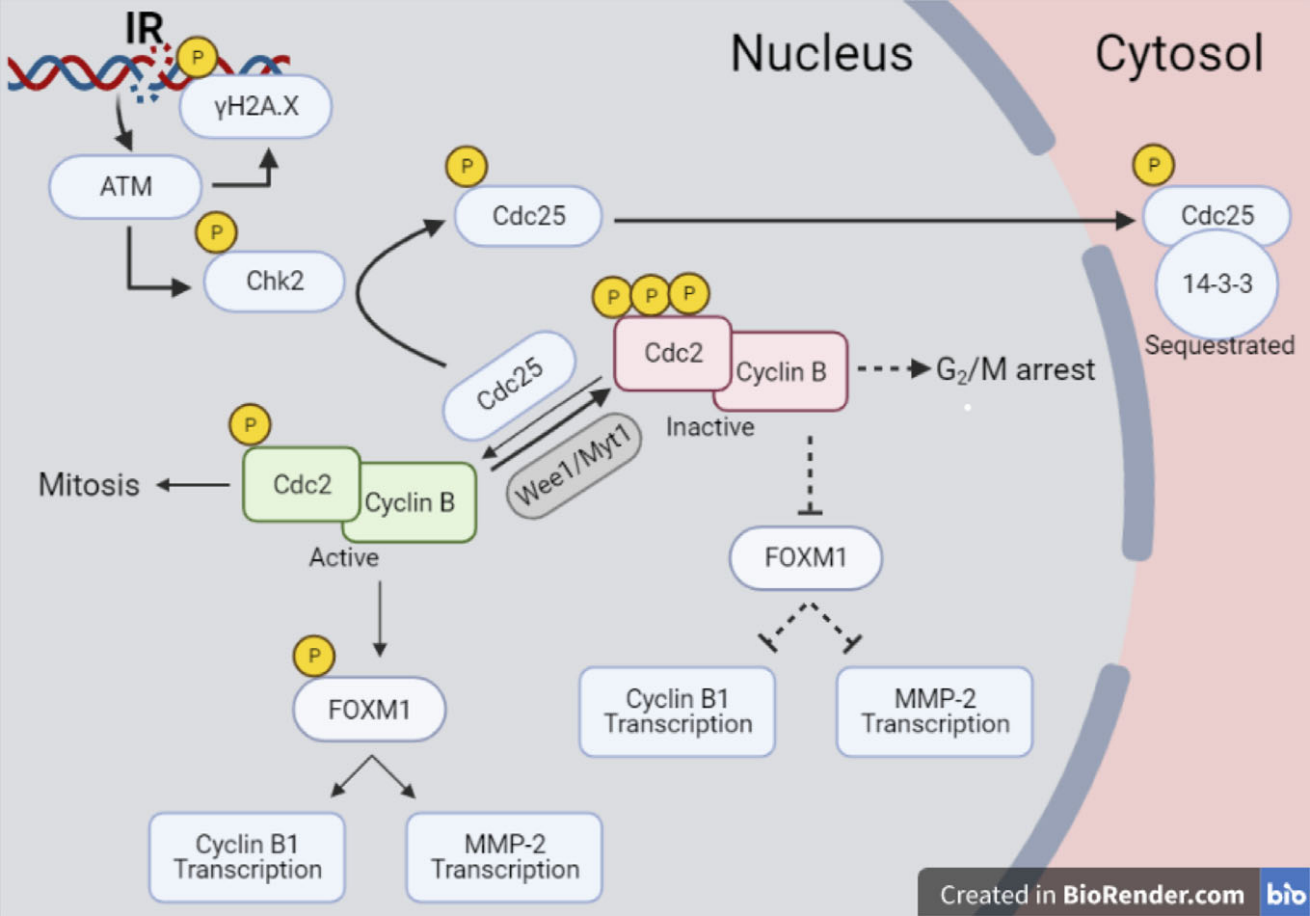
Pathway scheme of Caco-2 response to X-rays. Illustration with the proposed reconstruction of the chain of events started by radiation exposure of Caco-2 cells at a molecular level, starting from initial DNA damage and including several proteins involved in cell cycle regulation, particularly focusing on the G_2_/M transition. Illustration created with BioRender.com.

Finally, the new data set offers the chance to propose an interpretation of different results in the common scheme of specific pathways activated by radiation. In particular, despite the p53^null^ status of Caco-2, results presented in this work show a significant impact of radiation on such cell line in terms of short-term effects, as well as a reduced long-term replicative potential. This suggests focusing on a p53-independent pathway that can however lead to delays in cell cycle progression and possible associated arrest of cell-population growth. The proposed reconstruction of the chain of events started by radiation exposure at a molecular level is illustrated in **Figure 10**. As well known, one of the main critical targets of ionizing radiation is nuclear DNA, whose damage causes the activation of signaling pathways that lead to the regulation of DNA repair mechanisms and progression in the cell cycle. One of the early markers of DNA damage is the phosphorylation of histone H2AX: at our time points (certainly at 24 and 48 h), measured γ-H2AX signals correspond to forms of residual DNA damage, as the kinetics of DNA repair is known to be quicker (19). H2AX phosphorylation is induced by the ATM kinase in response to radiation. ATM induces as well the activation of Chk2, phosphorylating the T68 residue, and causing the subsequent phosphorylation of S216 residue of the Cdc25C phosphatase, which is exported to the cytosolic compartment and sequestrated by 14-3-3 proteins family. This mechanism drives specific molecules (*e.g.* WEE1, Myt1) to inactivate the Cdc2/CycB complex by hyperphosphorylation, causing a G_2_/M transition arrest (20). The inactivated Cdc2/CycB complex is not able to phosphorylate FoxM1 at S251, suppressing its transcriptional activity (21). In a recent study, it was demonstrated by means of ChIP-Seq analysis that FoxM1 is able to bind chromatin regions of a wide variety of genes, inducing their regulation (22, 23), thus playing an important role in different pathways including cell proliferation, migration, invasion, epithelial-to-mesenchymal transition. If this signaling cascade is inhibited in presence of a G_2_ arrest, FoxM1 cannot activate neither cell cycle regulator genes, such as CycB1, nor MMP-2.

The scheme of **Figure 10** allows to give a coherent interpretation of results reported in **Figures 8** and **9** and integrate them in the analysis of the other endpoints discussed in this work, overall suggesting the validity of this regulatory chain: increased levels of γ-H2AX are measured as a function of radiation dose, leading to increased levels of phosphorylated proteins induced by the initial DNA damage response (Chk2 and Cdc25C). The induction of the cell cycle arrest at the G_2_/M transition is confirmed by flow cytometry data (measured for both experimental protocols, data in **Figure 4** for cells undergoing the “Seed + Treat” protocol as those used for western blot analysis). As mentioned, the cause of such arrest (the inactivation of the Cdc2/CycB complex) has an impact on FoxM1 activity, which is measured to be down-regulated by radiation. The downstream signal of CycB1 is first increased with dose at 24 h after exposure, where flow cytometry data reveal the largest accumulation of cells in G_2_/M, and finally found to decrease at 48 h, this time following down-regulation of FoxM1. Within the same 48-h interval from the exposure, we also know from fluorescence microscopy images (**Figure 7**) that radiation is able to induce a higher mitotic instability, related to an incorrect regulation of G_2_ to M phase progression mediated by Cdc2/CycB1. This incorrect regulation can lead to a so-called mitotic catastrophe, evaluable with the premature chromatin condensation and the activation of death mechanisms *e.g.* apoptosis (24), which is also confirmed by flow cytometry data (**Figure 6**). Finally, down-regulation of FoxM1 also leads to the inhibition of MMP-2 activity (25, 26), confirmed by gelatin zymography (**Figure 8**).

Concluding, the collection of the results and the integrated analysis presented in this work deliver: i) a full characterization of the radiation response of Caco-2 cells, including how such response is affected by different experimental protocols. Starting from this characterization, Caco-2 cells can be further used as a peculiar colorectal cancer cell model, possibly extending this work to additional cell lines (*e.g.* HT-29 and DLD-1) identified as more aggressive colorectal cancer cell models; ii) a molecular characterization of mechanisms behind Caco-2 radiation response, that can as well be exploited in preclinical studies (as mentioned above, also comparing results for different CRC models) to identify possible targets to increase therapeutic effectiveness for CRC. In this latter framework, it is of note that FoxM1 appears to be a candidate target protein to address colorectal cancer resistance to one of the most chemotherapeutic drugs Fluoropyrimidine (5-Flourouracil, 5-FU), as suggested by new evidences: FoxM1 depletion has been associated with reduced CRC carcinogenesis and growth after exposure to carcinogens (27); resistance after drug treatment is known to be dependent on the p53 status of cells (9), but it is also modulated by FoxM1 (23), which makes the investigation of FoxM1 particularly interesting in Caco-2 cells (p53^null^) compared to colorectal cancer cell lines with different p53 status; FoxM1 is also involved in regulation of the cell microenvironment, *e.g.* regulating the promoters of matrix metalloproteases MMP-2 and MMP-9. MMP-2 activity and expression are strongly associated with advanced tumor stage or poor survival (28, 29).

In this research framework, this work sets the basis for future *in vitro* experimental studies [as well as for the development of computational models (30)] to develop new therapeutic strategies or explore synergistic effects in combined treatments (*e.g.* radiation, including the effect of fractionation as in a more realistic clinical setting, and other cytotoxic agents as chemotherapeutic drugs) using Caco-2 cell line [also, foreseeing the possibility of a 3D culture to better mimic the *in vivo* situation (31)] as a model for colorectal cancer.

## Data Availability Statement

The raw data supporting the conclusions of this article will be made available by the authors, without undue reservation.

## Author Contributions

IG, LL, and GB: conceived of the experiments. IG and LL: performed the experiments. IG, LL, MS, LAS, and GB: performed data analysis and data interpretation. IG, LL, and GB: wrote and edited the manuscript. MS, LAS, AO, and GB critically read the manuscript. All authors contributed to the article and approved the submitted version.

## Funding

This work was funded by the University of Pavia, Pavia, Italy.

## Conflict of Interest

The authors declare that the research was conducted in the absence of any commercial or financial relationships that could be construed as a potential conflict of interest.
